# The Causal Effects of Primary Biliary Cholangitis on Thyroid Dysfunction: A Two-Sample Mendelian Randomization Study

**DOI:** 10.3389/fgene.2021.791778

**Published:** 2021-12-10

**Authors:** Peng Huang, Yuqing Hou, Yixin Zou, Xiangyu Ye, Rongbin Yu, Sheng Yang

**Affiliations:** ^1^ Department of Epidemiology, Center for Global Health, School of Public Health, Nanjing Medical University, Nanjing, China; ^2^ Department of Biostatistics, Center for Global Health, School of Public Health, Nanjing Medical University, Nanjing, China

**Keywords:** thyroid dysfunction, hypothyroidism, thyroid cancer, two-sample Mendelian randomization, genome-wide association study

## Abstract

**Background:** Primary biliary cholangitis (PBC) is an autoimmune disease and is often accompanied by thyroid dysfunction. Understanding the potential causal relationship between PBC and thyroid dysfunction is helpful to explore the pathogenesis of PBC and to develop strategies for the prevention and treatment of PBC and its complications.

**Methods:** We used a two-sample Mendelian randomization (MR) method to estimate the potential causal effect of PBC on the risk of autoimmune thyroid disease (AITD), thyroid-stimulating hormone (TSH) and free thyroxine (FT4), hyperthyroidism, hypothyroidism, and thyroid cancer (TC) in the European population. We collected seven datasets of PBC and related traits to perform a series MR analysis and performed extensive sensitivity analyses to ensure the reliability of our results.

**Results:** Using a sensitivity analysis, we found that PBC was a risk factor for AITD, TSH, hypothyroidism, and TC with odds ratio (OR) of 1.002 (95% CI: 1.000–1.005, *p* = 0.042), 1.016 (95% CI: 1.006–1.027, *p* = 0.002), 1.068 (95% CI: 1.022–1.115, *p* = 0.003), and 1.106 (95% CI: 1.019–1.120, *p* = 0.042), respectively. Interestingly, using reverse-direction MR analysis, we also found that AITD had a significant potential causal association with PBC with an OR of 0.021 (*p* = 5.10E−4) and that the other two had no significant causal relation on PBC.

**Conclusion:** PBC causes thyroid dysfunction, specifically as AITD, mild hypothyroidism, and TC. The potential causal relationship between PBC and thyroid dysfunction provides a new direction for the etiology of PBC.

## Introduction

Primary biliary cholangitis (PBC) is an autoimmune cholestatic liver disease with a progressive disease (Lleo et al., 2017). Its prevalence and annual incidence rate are from 6.7 to 492 cases and from 0.7 to 49 cases per million inhabitants, respectively (Delgado et al., 2012). In addition, some population-based studies that investigate the incidence and prevalence of PBC are increasing year by year (Carey et al., 2015; Rosa et al., 2018; Lindor et al., 2019). Even worse, similar to other autoimmune diseases, the pathogenesis of PBC is complex and multifactorial, which results in no effective treatment for PBC. PBC can lead to cirrhosis, liver cancer, liver failure, and death within 10 years (Pratt, 2016; Younossi et al., 2019). With increasing prevalence and serious complications, it is worthy of our further study to explore the possible pathogenesis of PBC.

As known, up to 73% of PBC patients have extrahepatic manifestations (i.e., Sjogren’s syndrome, thyroid dysfunction, and systemic sclerosis) whose high incidence declines the quality of life (Chalifoux et al., 2017). Among them, thyroid dysfunction occurs in 5.6%–23.6% of PBC patients, which is obviously larger than that in the individuals without PBC (Huang and Liaw, 1995; Gershwin et al., 2005; Silveira et al., 2009; Chalifoux et al., 2017). Thyroid dysfunction results from excessive or insufficient production of thyroid hormone regulating human growth, neuron development, reproduction, and energy metabolism and may lead to various thyroid diseases such as hypothyroidism (Taylor et al., 2018). Studies have shown that some patients have symptoms of hypothyroidism and PBC symptoms simultaneously, and the incidence of hypothyroidism increases in patients with PBC (Elta et al., 1983). It is reported that levels of serum thyroid-stimulating hormone (TSH) and average serum free thyroxine (FT4) are higher in PBC patients (Schussler et al., 1978). The two kinds of hormone imbalance also occur in cirrhotic patients (Vincken et al., 2017; Punekar et al., 2018). In addition, patients with PBC, an autoimmune liver disease (AILD), are at higher risk for other autoimmune diseases, including autoimmune thyroid disease (AITD), Hashimoto’s thyroiditis, and Graves’s thyroiditis (Floreani et al., 2015; Suzuki et al., 2016; Zeng et al., 2020). However, the abovementioned relationships between PBC and thyroid dysfunction are obtained based on observational studies, in which reverse causality, selection bias, and especially unobserved confounding factors might mask true causal relationships. It is essential to further investigate the causal association underlying these correlations.

Mendelian randomization (MR) is widely used for causal inference in observational studies by treating single-nucleotide polymorphisms (SNPs) as instrumental variables (IVs) (Emdin et al., 2017). According to Mendel’s law of inheritance, alleles are transmitted randomly from parents to offspring during meiosis without interference from external factors (Reyna and Pickler, 1999). Therefore, MR has a natural advantage in determining causal relationships by removing the unobserved confounding. In addition, two-sample MR, requiring the exposure and outcome measured in independent but homogeneous samples, is accessible for the abundant resources and availability of summary statistics of genome-wide association studies (GWASs) (Evans and Davey Smith, 2015; Watanabe et al., 2019). MR is established on the basis that if a causal relationship exists between PBC and thyroid dysfunction, the SNP related to PBC will also be related to thyroid dysfunction through the occurrence of PBC, in which case the IVs only associate with PBC, and MR can help establish a causal relationship between PBC and thyroid function (Davey Smith and Hemani, 2014). Also, two-sample MR has been used to explore the causal relationship between thyroid function and breast cancer (Yuan et al., 2020), atrial fibrillation (Ellervik et al., 2019), and blood lipid profile (Wang et al., 2021).

Here, we comprehensively investigate the potential causal relationship between PBC and thyroid function. Specifically, we use seven large-scale GWAS summary statistics in the European population on PBC and thyroid indicators and disorders, including AITD, TSH, FT4, hyperthyroidism, hypothyroidism, and thyroid cancer (TC), to perform a series of two-sample MR. Furthermore, we also perform several sensitivity analyses, including the heterogeneity test, pleiotropy test, leave-one-out (LOO) test, and reverse-direction MR analyses to ensure the reliability of our results.

## Methods

### Data Collection

We collected seven datasets on PBC and related traits, including one PBC dataset (Cordell et al., 2015), one AITD dataset (including both Hashimoto’s thyroiditis and Graves’ disease) (Glanville et al., 2021), four datasets (for TSH, FT4, hyperthyroidism, and hypothyroidism) from The ThyroidOmics Consortium (Teumer et al., 2018), and one TC dataset (Rashkin et al., 2020). Specifically, PBC dataset contained 13,239 individuals (Prev. = 0.209); AITD dataset contained 324,933 individuals (Prev. = 0.003); TSH dataset contained 54,288 individuals; FT4 dataset contained 49,269 individuals; hyperthyroidism dataset included 51,668 individuals (Prev. = 0.626); hypothyroidism dataset included 53,241 individuals (Prev. = 0.036); and TC dataset included 411,112 individuals (Prev. = 0.002). All summary data came from the European population. Then, we filter out SNPs 1) with INFO < 0.6, 2) with minor allele fraction (MAF) <0.01, 3) with palindrome alleles, and 4) whose odds ratio (OR) was larger or smaller than the mean ± 3 SD. Finally, we obtained 1,134,141, 9,390,112, 7,666,442, 7,138,715, 7,138,916, 7,191,562, and 9,291,956 SNPs for the seven traits. In addition, we used linkage disequilibrium score regression (LDSC) (v1.0.1) to estimate heritability (*h* ^2^) for each dataset. We set the population prevalence (*-*-pop-prev) for the five diseases (PBC, AITD, hyperthyroidism, hypothyroidism, and TC) to 0.209, 0.003, 0.036, 0.063, and 0.002 to estimate liability heritability. The detailed information of the seven datasets is shown in Supplementary Table S1.

### Instrumental Variable Selections

**FIGURE 1:**
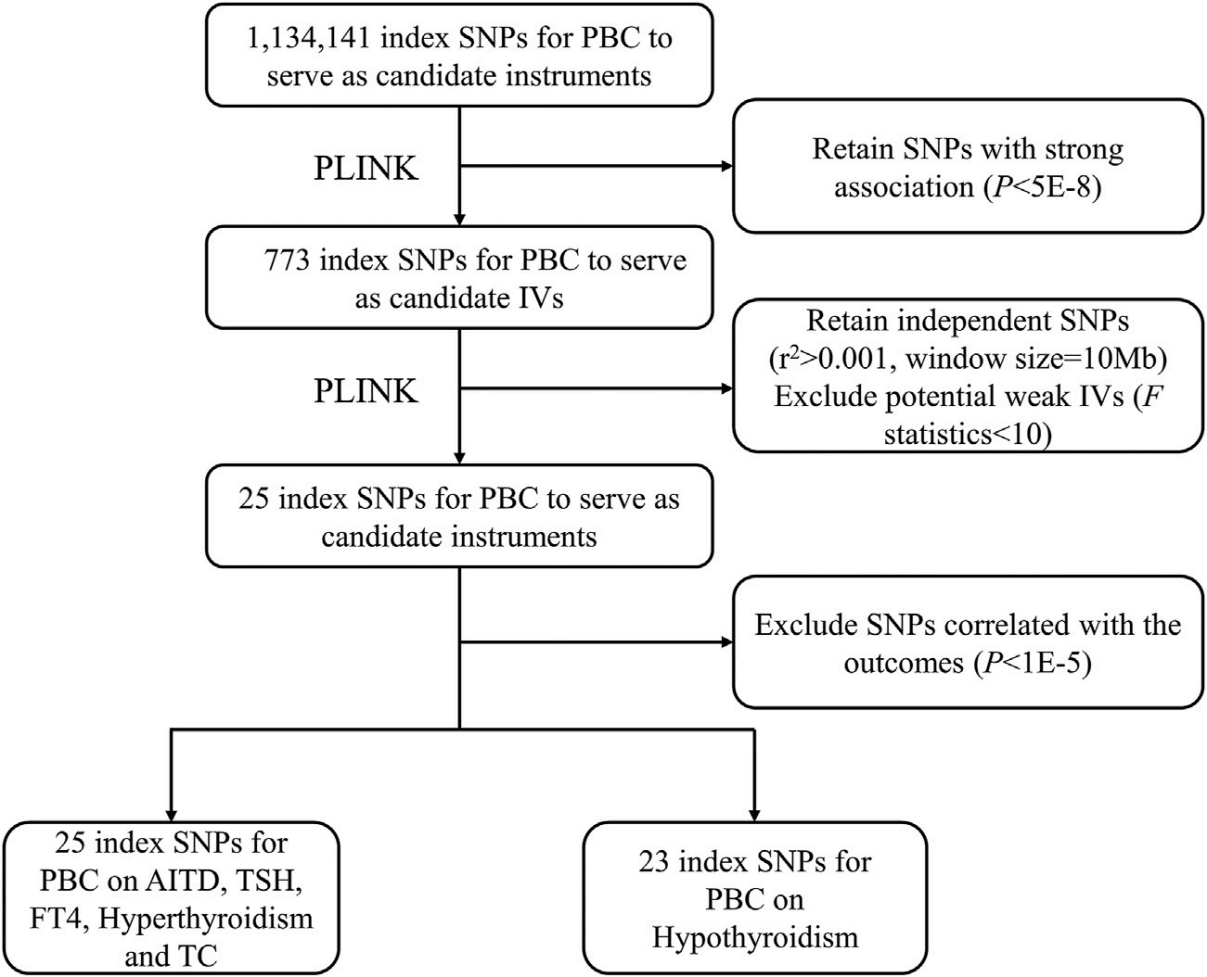
The flowchart for IV selection. The flowchart shows the selection process of PBC IVs to estimate the causal effects on AITD, TSH, FT4, hyperthyroidism, hypothyroidism, and TC. First, we use *p* < 5.00E−8 to select index SNPs to ensure that they strongly associate with PBC. Second, we use r^2^ > 0.001 in the range of 10 Mb to select independent index SNPs. We treat the EUR of 1000 Genomes Project as the reference panel. The first two steps are completed by PLINK. Finally, we obtain 25 IVs on AITD, TSH, FT4, hyperthyroidism, and TC and 23 IVs on hypothyroidism. IV, instrumental variable; PBC, primary biliary cholangitis; AITD, autoimmune thyroid disease; TSH, thyroid-stimulating hormone; FT4, free thyroxine; TC, thyroid cancer; SNP, single-nucleotide polymorphism.

A crucial step of MR was to choose appropriate genetic variants to serve as valid IVs for PBC. Based on the above datasets, we followed the strict screening procedures in other previous MR studies to select IVs (Zeng et al., 2019; Dong et al., 2021) (Figure 1). First, we retained 773 variants for PBC with a *p*-value smaller than 5.00E−8. Second, we removed 748 highly correlated variants with r^2^ greater than 0.001 in the range of 10 Mb. In addition, we ensured that each alternative SNP selected as IV was strongly associated with PBC. According to the previous research (Zeng et al., 2019), we calculated the *F* statistic to find weak IVs, and no variant was excluded with a minimum *F* statistic of 30.16. Finally, we only kept a total of 25 independent candidate IVs to study the causal relationship between PBC and the other six traits. The details of these IVs are shown in Supplementary Table S2.

We carried out three two-sample MR analyses, including fixed-effects and random-effects inverse variance weighting (IVW), MR-Egger, and weighted median (WM) methods, to estimate the potential causal effect of PBC on the six traits (Bowden et al., 2015; Bowden et al., 2016a). Without consideration for the intercept term, IVW regarded the reciprocal of the outcome variance (the square of SE) as the weight. Under the assumption of IVW, we consider that IVs are not pleiotropic. Therefore, we must ensure that these IVs are not pleiotropic when using the IVW method; otherwise, the results were biased (Bowden et al., 2015). Different from IVW, MR-Egger used an intercept term to measure the horizontal pleiotropy between these IVs (Bowden et al., 2016b). The WM method assumed that variables that account for at least 50% of the total IVs were valid, so the causal effects can be estimated consistently (Bowden et al., 2016a).

### Sensitivity Analysis

Following methods in previous studies (Noyce et al., 2017; Zeng and Zhou, 2019), we performed a sensitivity analysis to evaluate the potential violations of the model assumptions in the MR analysis: 1) heterogeneity test, 2) pleiotropic test, and 3) LOO test. First, heterogeneity analysis estimates the heterogeneity between IVs. If the heterogeneity exists, it would be hard to combine the IVs directly. Second, if IVs can directly affect the results without exposure factors, then they violate the idea of MR; that is, the level of pleiotropy in the test results will lead to serious deviations in MR (Hemani et al., 2018; Ong and MacGregor, 2019). We use MR pleiotropy residual sum and outlier (MR-PRESSO) to find outliers and test the level of pleiotropy. For more verification, we still use the MR-Egger intercept to test the pleiotropy. Finally, the LOO test refers to gradually removing each SNP, calculating the meta effect of the remaining SNPs, and observing whether the result significantly changed after removing each SNP. Ideally, no significant difference meant a robust result (Noyce et al., 2017). All the analyses are performed by R software (v4.1.1). Specially, we used TwoSampleMR R package (v0.5.6) to perform MR analysis. The statistical significance level was set to 0.05 throughout our study.

### Reverse-Direction Mendelian Randomization Analyses

We also performed reverse-direction MR to assess potential reverse causal effects of AITD, TSH, FT4, hyperthyroidism, hypothyroidism, and TC on PBC. Following methods in previous literature (Savage et al., 2018; Dong et al., 2021), for each exposure, we used the clumping algorithm in PLINK (Chang et al., 2015) to select independent SNPs for each trait (r^2^ threshold = 0.001, window size = 10 Mb, and *p* < 5.00E−8). Finally, we obtained two IVs for AITD, 38 IVs for TSH, 17 IVs for FT4, 7 IVs for hyperthyroidism, 6 IVs for hypothyroidism, and 3 IVs for TC. We used these IVs of six traits to perform reverse causal inferences on PBC to assess potential reverse causal effects. The reverse-direction MR analysis process was the same as previously described.

## Results

### Summary of Genome-Wide Association Study Data

We estimated the heritability for each trait. Specifically, the genetic inflation factor (*λ*_gc_) of PBC is 1.050 (LDSC intercept: 1.003); *λ*_gc_ of AITD is 0.999 (LDSC intercept: 0.999); *λ*_gc_ of TSH is 1.077 (LDSC intercept: 1.035); *λ*_gc_ of FT4 is 1.111 (LDSC intercept: 1.014); *λ*_gc_ of hyperthyroidism is 1.029 (LDSC intercept: 1.113); *λ*_gc_ of hypothyroidism is 1.044 (LDSC intercept: 1.083); and *λ*_gc_ of TC is 1.008 (LDSC intercept: 0.999). With the use of GWAS summary statistics and 1000 Genomes Project (1000 GP) EUR reference panel, the SNP-based liability heritability for PBC, AITD, and TC is 595.942, 0.012, and 0.103, respectively. The observed heritability for PBC, AITD, TSH, FT4, and TC is 0.378, 0.003, 0.125, 0.152, and 0.002, respectively (Supplementary Table S1). We used the Manhattan plot to show the GWAS results for seven traits (Supplementary Figure S1).

### Mendelian Randomization Analysis

**FIGURE 2:**
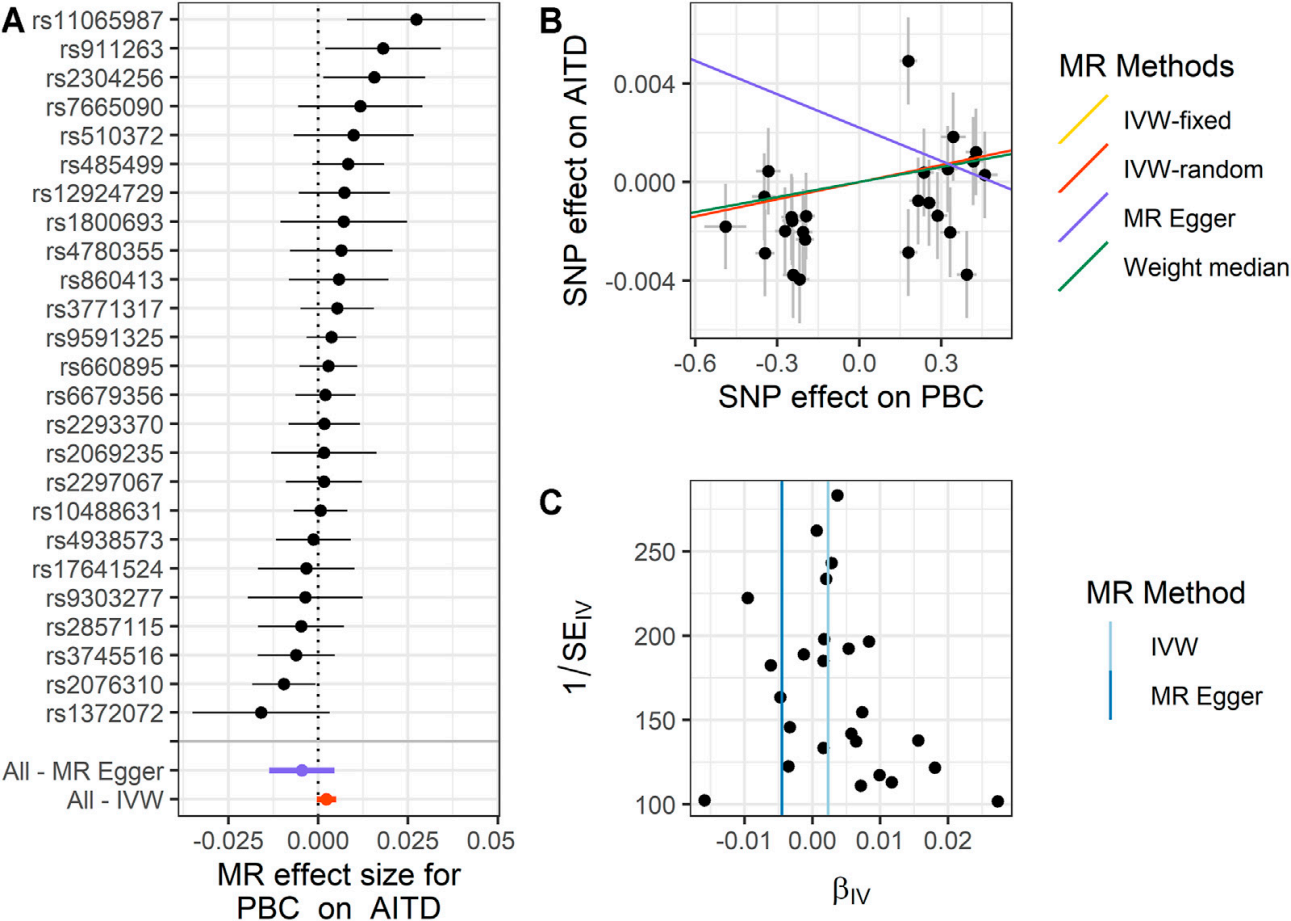
Summary of the MR analysis for PBC on AITD. **(A)** MR effect size of each IV, MR-Egger, and IVW. **(B)** The scatter plot of causal effects of PBC on AITD. We use vertical and horizontal black lines to show 95% CI of the estimated effect of IVs on PBC (x-axis) and that on AITD (y-axis), respectively. We use the red line to show the IVW random-effects model. **(C)** The funnel plot of the causal effect of PBC on AITD. Each point represents the estimated causal effect of each IV. The vertical dark blue line represents the causal effect estimate obtained using the MR-Egger method; the light blue line represents the causal effect estimate obtained using the IVW method. MR, Mendelian randomization; PBC, primary biliary cholangitis; AITD, autoimmune thyroid disease; IV, instrumental variable; IVW, inverse variance weighting.

**FIGURE 3:**
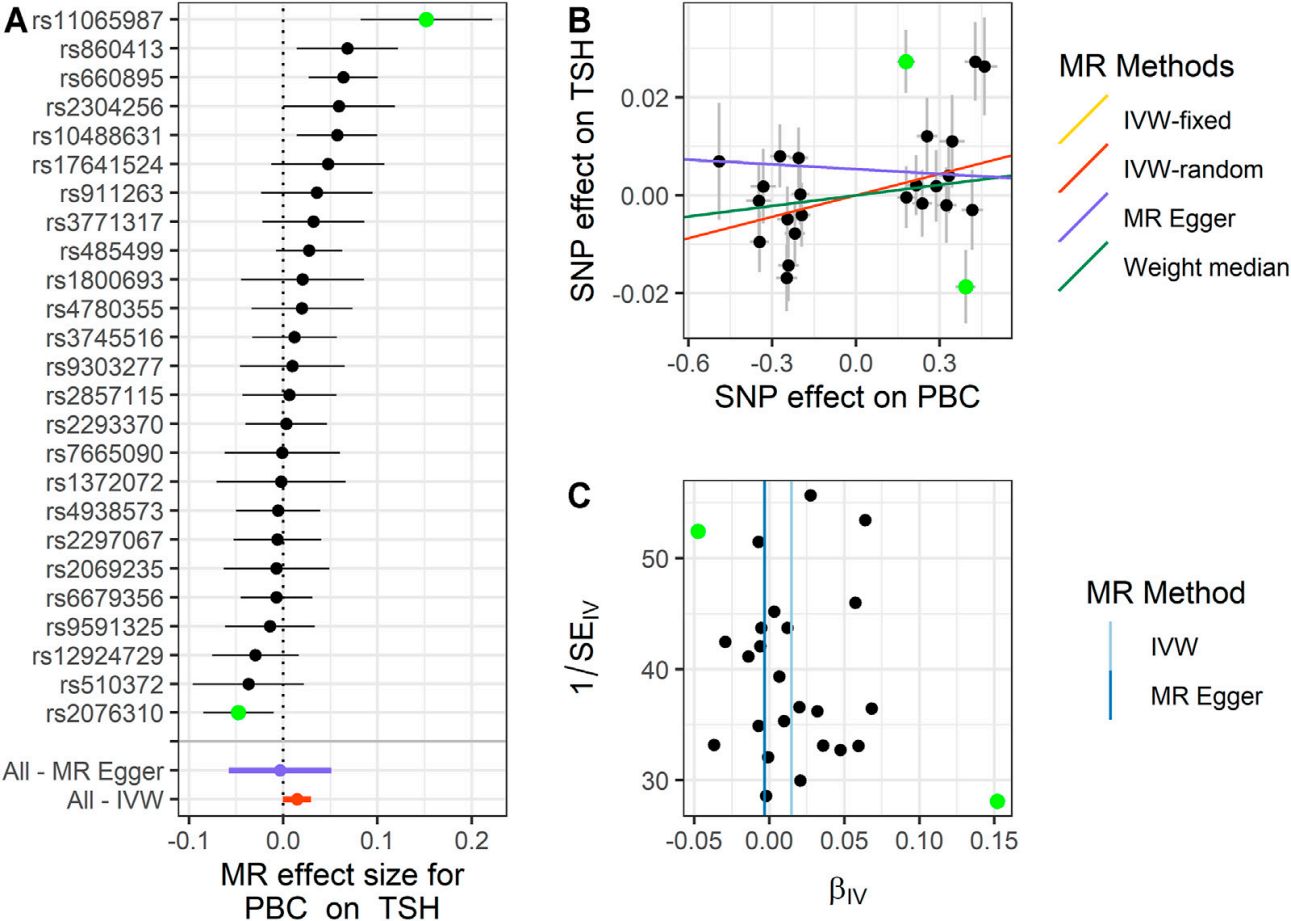
Summary of the MR analysis for PBC on TSH. **(A)** MR effect size of each IV, MR-Egger, and IVW. **(B)** The scatter plot of causal effects of PBC on TSH. We use vertical and horizontal black lines to show 95% CI of the estimated effect of IVs on PBC (x-axis) and that on TSH (y-axis), respectively. We use the red line to show the IVW random-effects model. The potential SNP outlier (rs11065987 and rs2076310) is highlighted in green. **(C)** The funnel plot of the causal effect of PBC on TSH. Each point represents the estimated causal effect of each IV. The vertical dark blue line represents the causal effect estimate obtained using the MR-Egger method; the light blue line represents the causal effect estimate obtained using the IVW method. The potential SNP outlier (rs11065987 and rs2076310) is highlighted in green. MR, Mendelian randomization; PBC, primary biliary cholangitis; TSH, thyroid-stimulating hormone; IV, instrumental variable; IVW, inverse variance weighting; SNP, single-nucleotide polymorphism.

**FIGURE 4:**
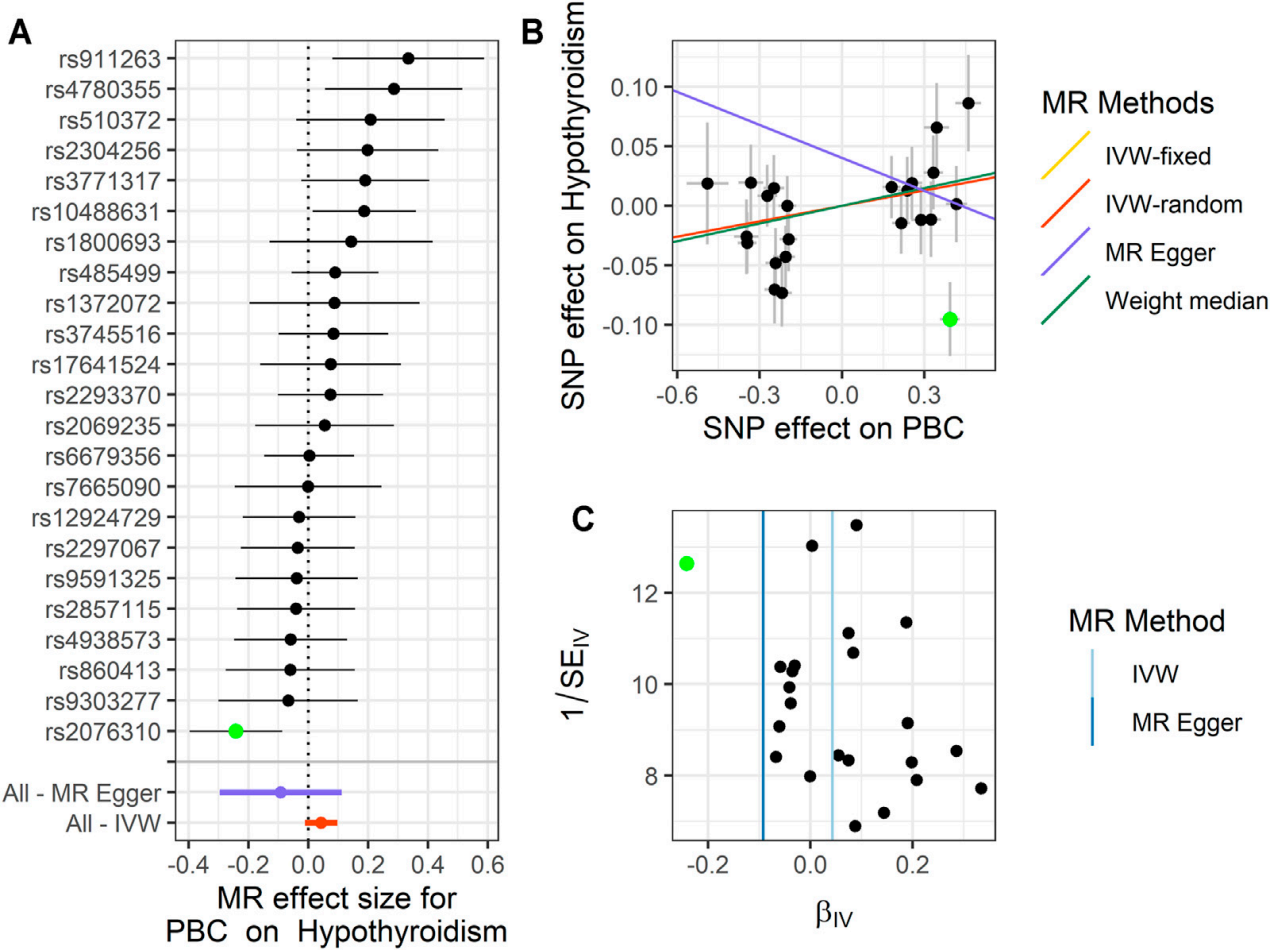
Summary of the MR analysis for PBC on hypothyroidism. **(A)** MR effect size of each IV, MR-Egger, and IVW. **(B)** The scatter plot of causal effects of PBC on hypothyroidism. We use vertical and horizontal black lines to show 95% CI of the estimated effect of IVs on PBC (x-axis) and that on hypothyroidism (y-axis), respectively. We use the red line to show the IVW random-effects model. The potential SNP outliers (rs2076310) are highlighted in green. **(C)** The funnel plot of the causal effect of PBC on hypothyroidism. Each point represents the estimated causal effect of each IV. The vertical dark blue line represents the causal effect estimate obtained using the MR-Egger method; the light blue line represents the causal effect estimate obtained using the IVW method. The potential outliers (rs2076310) are highlighted in green. MR, Mendelian randomization; PBC, primary biliary cholangitis; IV, instrumental variable; IVW, inverse variance weighting; SNP, single-nucleotide polymorphism.

We performed MR analysis on the IVs of PBC selected on six traits. Except for hypothyroidism, which only had 23 IVs, the other five traits were all 25 IVs. Based on different assumptions, we estimated the potential causal effects of four models, including IVW (fixed- and random-effects models), MR-Egger, and WM. And we use forest plots to show the causal relationship of a single IV in each trait, scatter plots to show the overall fitting causal effects between PBC and the traits, and funnel plots to show the relationship between the effect of the MR model and the effect of each SNP (Figures 2–4; Supplementary Figures S2–S4, Supplementary Tables S3–S8). For the causal effect for the six traits, we should use the result of the sensitivity analysis to determine whether the analysis result is significant.

For PBC on AITD, we observe a positive causal effect. The estimated OR from fixed-effects IVW method is 1.002 (95% CI: 1.000–1.005, *p* = 0.042). However, the result of the random-effects IVW method (OR = 1.002, 95% CI: 0.999–1.005, *p* = 0.092) is similar to that of fixed-effects IVW, but it is not significant. The result of WM (OR = 1.002, 95% CI: 1.000–1.005, *p* = 0.196) and MR-Egger (OR = 0.995, 95% CI: 0.987–1.005, *p* = 0.339) is similar to that of the random-effects IVW method. The above results indicate that AITD would increase with the increase of PBC risk. The details are shown in Figure 2 and Supplementary Table S3.

For PBC on TSH, we also observe a positive causal effect. The estimated OR from fixed-effects IVW method is 1.015 (95% CI: 1.005–1.025, *p* = 0.003). The result of the random-effects IVW method (OR = 1.015, 95% CI: 1.000–1.030, *p* = 0.056) is similar to that of fixed-effects IVW, but it is not significant. The result of WM (OR = 1.007, 95% CI: 0.991–1.024, *p* = 0.362) and MR-Egger (OR = 0.997, 95% CI: 0.944–1.052, *p* = 0.908) is similar to that of the random-effects IVW method. The above results indicate that TSH would increase with the increase of PBC risk. The details are shown in Figure 3 and Supplementary Table S4.

For PBC on FT4, we failed to define a significant causal effect. The estimated OR from fixed-effects IVW method is 1.005 (95% CI: 0.994–1.015, *p* = 0.375). And the result of the random-effects IVW method (OR = 1.005, 95% CI: 0.994–1.015, *p* = 0.380) is similar to that of fixed-effects IVW. The result of WM (OR = 1.007, 95% CI: 0.992–1.022, *p* = 0.372) and MR-Egger (OR = 0.997, 95% CI: 0.960–1.036, *p* = 0.893) is similar to the above conclusion. The above results indicate that FT4 would increase with the increase of PBC risk, but none of them is significant. The details are shown in Supplementary Figure S2 and Supplementary Table S5.

For PBC on hyperthyroidism, we failed to define any significant causal effect using the four models. The estimated OR from fixed-effects IVW method is 0.984 (95% CI: 0.937–1.034, *p* = 0.534). And the result of the random-effects IVW method (OR = 0.984, 95% CI: 0.938–1.034, *p* = 0.524) is not significant, which is similar to that of fixed-effects IVW. The results of WM (OR = 1.007, 95% CI: 0.939–1.081, *p* = 0.836) and MR-Egger (OR = 0.933, 95% CI: 0.779–1.117, *p* = 0.457) are both not significant. The details are shown in Supplementary Figure S3 and Supplementary Table S6.

For PBC on hypothyroidism, in these 23 IVs, we observed the positive causal effect of PBC on hypothyroidism. Note that we only define the significant result from fixed-effects IVW method (OR = 1.044, 95% CI: 1.001–1.089, *p* = 0.044), rather than the result of the random-effects IVW method (OR = 1.044, 95% CI: 0.989–1.103, *p* = 0.122), the result of WM (OR = 1.051, 95% CI: 0.987–1.119, *p* = 0.116), and the result of MR-Egger (OR = 0.912, 95% CI: 0.744–1.118, *p* = 0.385). We should use the result of the sensitivity analysis to check for the outliers and determine whether the analysis result is representative. The details are shown in Figure 4 and Supplementary Table S7.

For PBC on TC, the result of PBC on TC is similar to that of hypothyroidism. The only significant result was from fixed-effects IVW method (OR = 1.106, 95% CI: 1.019–1.120, *p* = 0.042) rather than the result of the random-effects IVW method (OR = 1.106, 95% CI: 0.990–1.235, *p* = 0.074), the result of WM (OR = 1.137, 95% CI: 0.998–1.295, *p* = 0.054), and the result of MR-Egger (OR = 1.243, 95% CI: 0.836–1.850, *p* = 0.294). The details are shown in Supplementary Figure S4 and Supplementary Table S8.

### Sensitivity Analyses

We performed extensive sensitivity analyses to validate the results of the MR analysis, which are mainly of heterogeneity analysis and pleiotropic analysis on these IVs. We performed LOO analysis only for the significant causal effect. Our purpose was to explore whether the results obtained were robust, whether there was potential bias (such as pleiotropy and data heterogeneity), and whether there was a certain IV that seriously affects the outcome variable.

First, we conducted a heterogeneity analysis. Based on IVW, we found that TSH, hypothyroidism, and TC were heterogeneous as compared with AITD, FT4, and hypothyroidism. The *P*_*Q*_ of TSH, hyperthyroidism, and TC was 1.71E−4, 0.021, and 0.007, respectively. The *P*_*Q*_ of the remaining three traits was larger than 0.05. In order to reduce the heterogeneity, we chose to perform MR-PRESSO analysis to find and eliminate the outliers and can also test the pleiotropy of IVs.

Next, we performed MR-PRESSO analysis and LOO test to ensure the validation for MR analysis. For TSH, we found rs11065987 (beta = 0.027, *p* = 2.30E−5) and rs2076310 (beta = −0.019, *p* = 0.013) might be outliers that have affected the causal effect of IVs. After they were removed, the *p*-value of MR-PRESSO Global test changed from 0.001 to 0.098, which indicated that the pleiotropy was eliminated, and the *P*_*Q*_ of TSH was also changed to 0.089, indicating that the heterogeneity has been eliminated. The result of the LOO test was significant for rs2076310 (*P*_*LOO*_ = 0.008) and not significant for rs11065987 (*P*_*LOO*_ = 0.078). Therefore, we choose the result of the fixed-effects IVW method after removing the outliers as the significant causal effect for PBC on TSH. For hypothyroidism, we define that rs2076310 (beta = −0.095, *p* = 0.002) might be an outlier that has affected the causal effect of IVs. After it was removed, the *p*-value of MR-PRESSO Global test changed from 0.022 to 0.345, which indicated that the pleiotropy has been eliminated, and the *P*_*Q*_ of hypothyroidism was also changed to 0.321, indicating that the heterogeneity has been eliminated. The result of the LOO test for this outlier is the same as the above result (*P*_*LOO*_ *=* 0.005). Therefore, we chose the result of the fixed-effects IVW method after removing the outlier as the significant causal effect of PBC on hypothyroidism. And for TC, we did not identify any outliers.

Finally, we used the MR-Egger intercept to estimate pleiotropy. We defined no significant pleiotropy in six potential causal relationships. After the outliers were removed, the *p*-value of the MR-Egger intercept increased.

To sum up, we used the result from the fixed-effects IVW method to represent the causal effect of PBC on AITD (OR = 1.002, 95% CI: 1.000–1.005, *p* = 0.042); the result from fixed-effects IVW method with outlier excluded was used to represent the causal effect of PBC on TSH (OR = 1.016, 95% CI: 1.006–1.027, *p* = 0.002); and the result from fixed-effects IVW method with outlier excluded was used to represent the causal effect of PBC on hypothyroidism (OR = 1.068, 95% CI: 1.022–1.115, *p* = 0.003). The remaining three traits were not significant.

### Reverse-Direction Mendelian Randomization Analysis

In order to identify potential confounding factors that mislead the direction of causal effects, we performed reverse-direction MR (Supplementary Figures S5–10). We found that AITD and TC have a significant potential causal association with PBC with the random-effects IVW method, while the causal effects for TSH, FT4, hyperthyroidism, and hypothyroidism on PBC are not significant. Specifically, using the random-effects IVW method, the estimated OR for AITD and TC on PBC is 0.021 (*p* = 5.10E−4) and 1.026 (*p* = 0.011), respectively (Supplementary Tables S3, S8). Note that the results might be not reliable for the small number of IVs (Dong et al., 2021).

## Discussion

Here, we performed a comprehensive two-sample MR analysis to illustrate the potential causality between PBC and thyroid dysfunction. After a series of sensitivity analyses, we found that PBC significantly results in the occurrence of AITD (OR=1.002) and hypothyroidism (OR = 1.068) and that PBC significantly causes the increase of TSH level (OR = 1.016). Our findings provided an exploration direction for the occurrence of thyroid dysfunction in PBC patients, contributed to the treatment of thyroid diseases in PBC patients, and improved the quality of life for PBC patients. As expected, our results are consistent with previous observational population-based studies. For the potential causal relation of hypothyroidism, emerging evidences indicate that PBC is often with the occurrence of AITD (Crowe et al., 1980; Floreani et al., 2015; Patil et al., 2021) and hypothyroidism (Crowe et al., 1980; Elta et al., 1983) and the increase of TSH, one of the main signs of hypothyroidism (Patil et al., 2021).

For PBC on AITD, we define that PBC and AITD might be mutual cause-and-effect factors in both MR and reverse-direction MR analyses. Consistent with our findings, emerging epidemiological studies have shown that genetic components are important in the pathogenesis of Hashimoto’s thyroiditis (Paknys et al., 2009). The occurrence of PBC and AITD might be caused by environmental and genetic factors, such as intestinal flora (Fenneman et al., 2020), estrogen (Qin et al., 2018), gene-mediated immunodeficiency, and synergy between each other (Milette et al., 2019).

For PBC on hypothyroidism, Garber et al. also showed that PBC causes mild hypothyroidism, manifesting as only increasing TSH and normal FT4 levels (Garber et al., 2012). This finding is consistent with our results, that is, significant causal relation of PBC on TSH and insignificant causal relation of PBC on FT4.

There are several assumptions for the causality for PBC on hypothyroidism. One is the interaction between thyroid hormones and the liver (Salata et al., 1985). Liver damage caused by PBC can lead to changes in the expression of the enzyme D3 that controls the activity of thyroid hormones (Gilgenkrantz and Collin de l'Hortet, 2018), which can lead to a decrease in the accumulation of active thyroid hormones (Elbers et al., 2016), can trigger hypothalamic–pituitary–thyroid regulation disorders, and can increase TSH, leading to hypothyroidism. The second is that PBC cholestasis decreases Y protein, which in turn leads to hypothyroidism (Ariza et al., 1984). Protein Y is a type of protein that is distributed in the liver and promotes the absorption of thyroid hormones by the liver; the decrease of protein Y makes the liver speed up the circulation of thyroid hormones and reduce the free thyroid hormones in the blood (Reyes et al., 1971), leading to hypothalamus–pituitary–thyroid disorders, increase in TSH, and appearance of symptoms of hypothyroidism.

For the potential causal relation for PBC on TC, few studies have reported the association between PBC and TC. We assume that PBC causes thyroid dysfunction (such as hypothyroidism and thyroiditis), which eventually progresses to TC (dos Santos Silva and Swerdlow, 1993; Pacini et al., 2012). Studies have shown that AITD is one of the risk factors for TC, and elevated TSH levels and thyroid autoimmune characteristics are defined as independent risk factors for TC (Ferrari et al., 2020). Studies have also shown that thyroid tumors mainly exhibit hypothyroidism-like symptoms and that hypothyroidism may be the basis for most TCs (Hernandez et al., 2021). Our research is consistent with previous findings and explanations.

Our research also has some limitations. First, MR analysis cannot rule out the influence of hidden and unknown confounding factors, and we cannot completely rule out the association of IVs to confounding factors. This makes the assumptions of IVs strict and demanding. Especially weak IVs should be considered in the research. Second, MR analysis only provides directions for the etiology and progress of PBC and thyroid dysfunction, which lacks the biological mechanism behind the potential causal relationship. Last, the populations of the data we analyzed are all of European descent, the final results are limited by the genes of different races, and the results may not be very applicable to Asian populations.

In conclusion, our findings show that PBC can cause thyroid dysfunction, specifically as AITD, mild hypothyroidism, and TC. The potential causal relationship between PBC and thyroid dysfunction provides a new direction for the study of the etiology and progress of PBC.

## Data Availability

Publicly available datasets were analyzed in this study. These data can be found here: (https://www.immunobase.org/downloads/protected_data/GWAS_Data/hg19_gwas_pbc_cordell_4_20_0.tab.gz; https://transfer.sysepi.medizin.uni-greifswald.de/thyroidomics/datasets/; http://ftp.ebi.ac.uk/pub/databases/gwas/summary_statistics/GCST90014001-GCST90015000/GCST90014440/GCST90014440_buildGRCh37.tsv; https://github.com/Wittelab/pancancer_pleiotropy.
