# Supplementary figures and images for: The Causal Effects of Primary Biliary Cholangitis on Thyroid Dysfunction: A Two-Sample Mendelian Randomization Study
Source: Front Genet. 2021 Dec 10;12:791778. doi: 10.3389/fgene.2021.791778 (PMC8703001; doi:10.3389/fgene.2021.791778)

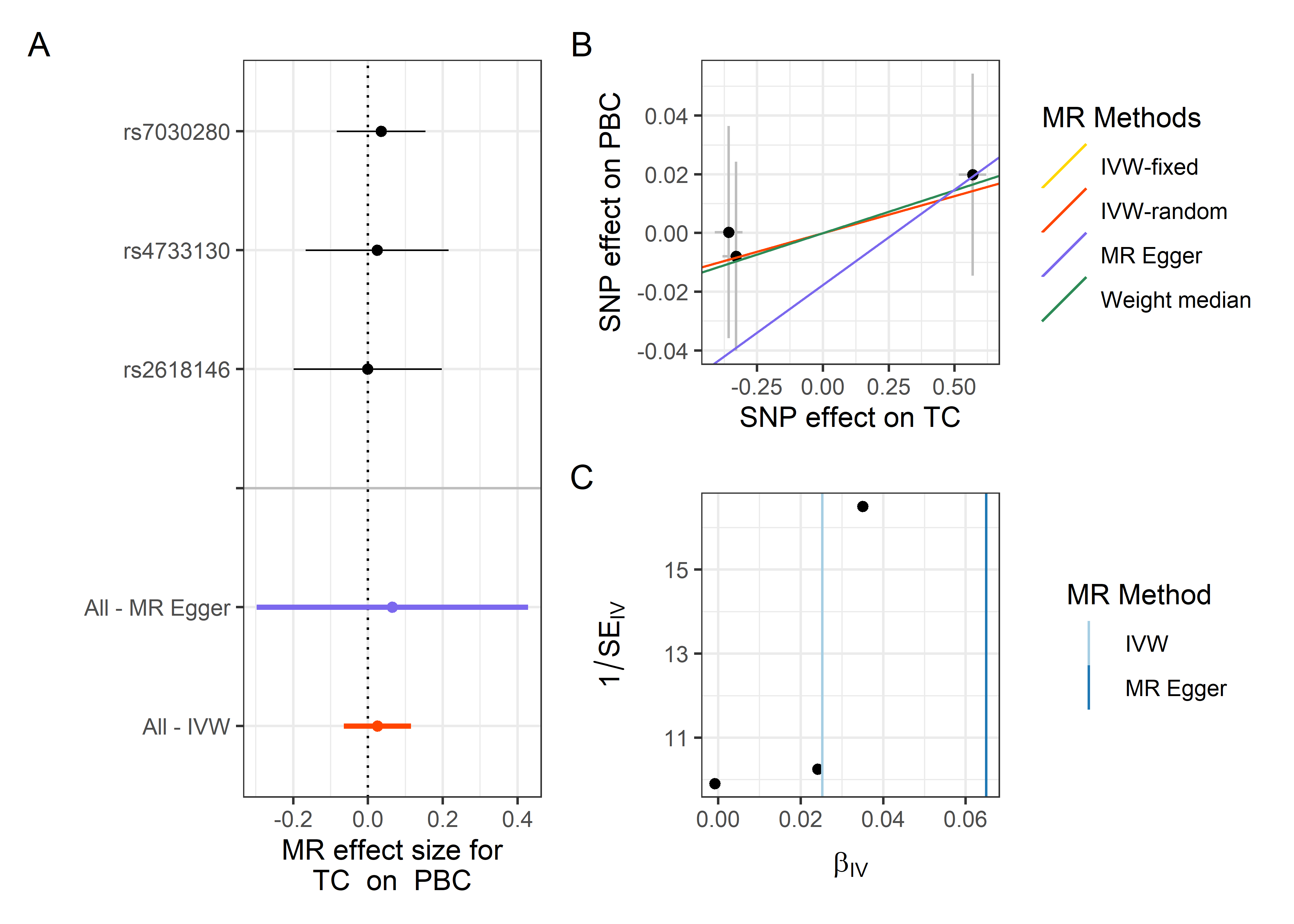

Supplement: Supplementary file 1 [file DataSheet1.ZIP › Supplementary figures/Fig.S10.tif]

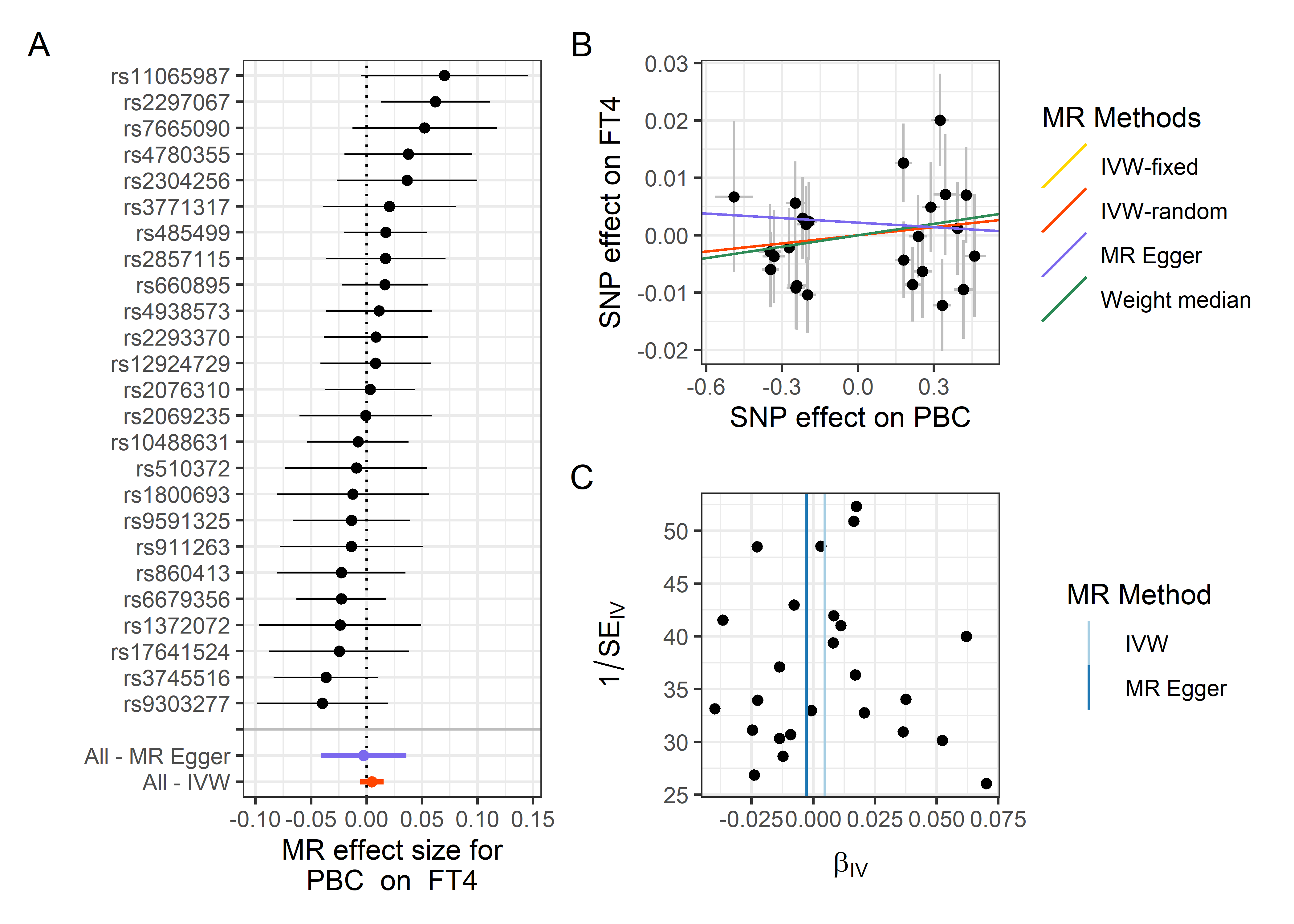

Supplement: Supplementary file 1 [file DataSheet1.ZIP › Supplementary figures/Fig.S2.tif]

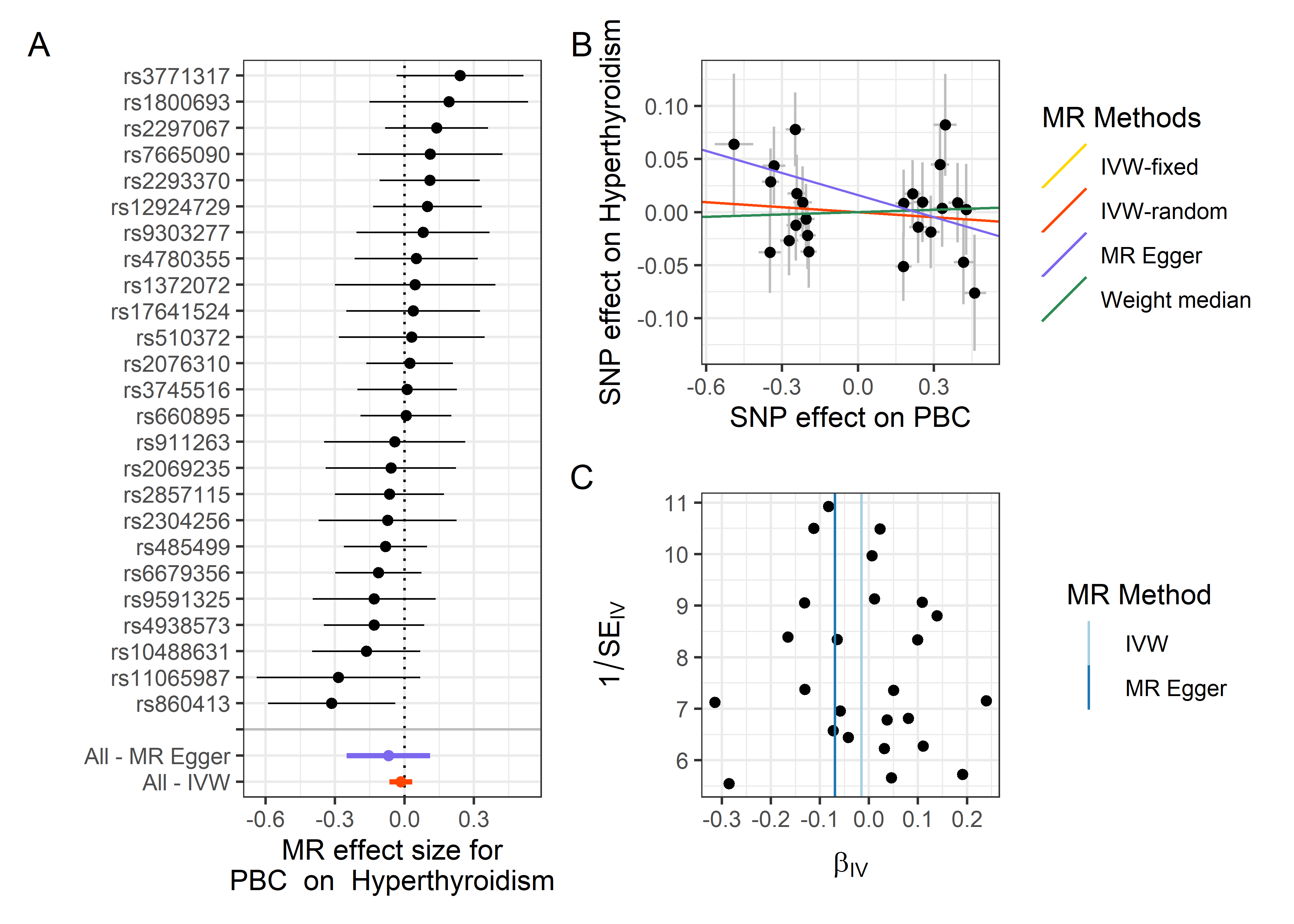

Supplement: Supplementary file 1 [file DataSheet1.ZIP › Supplementary figures/Fig.S3.tif]

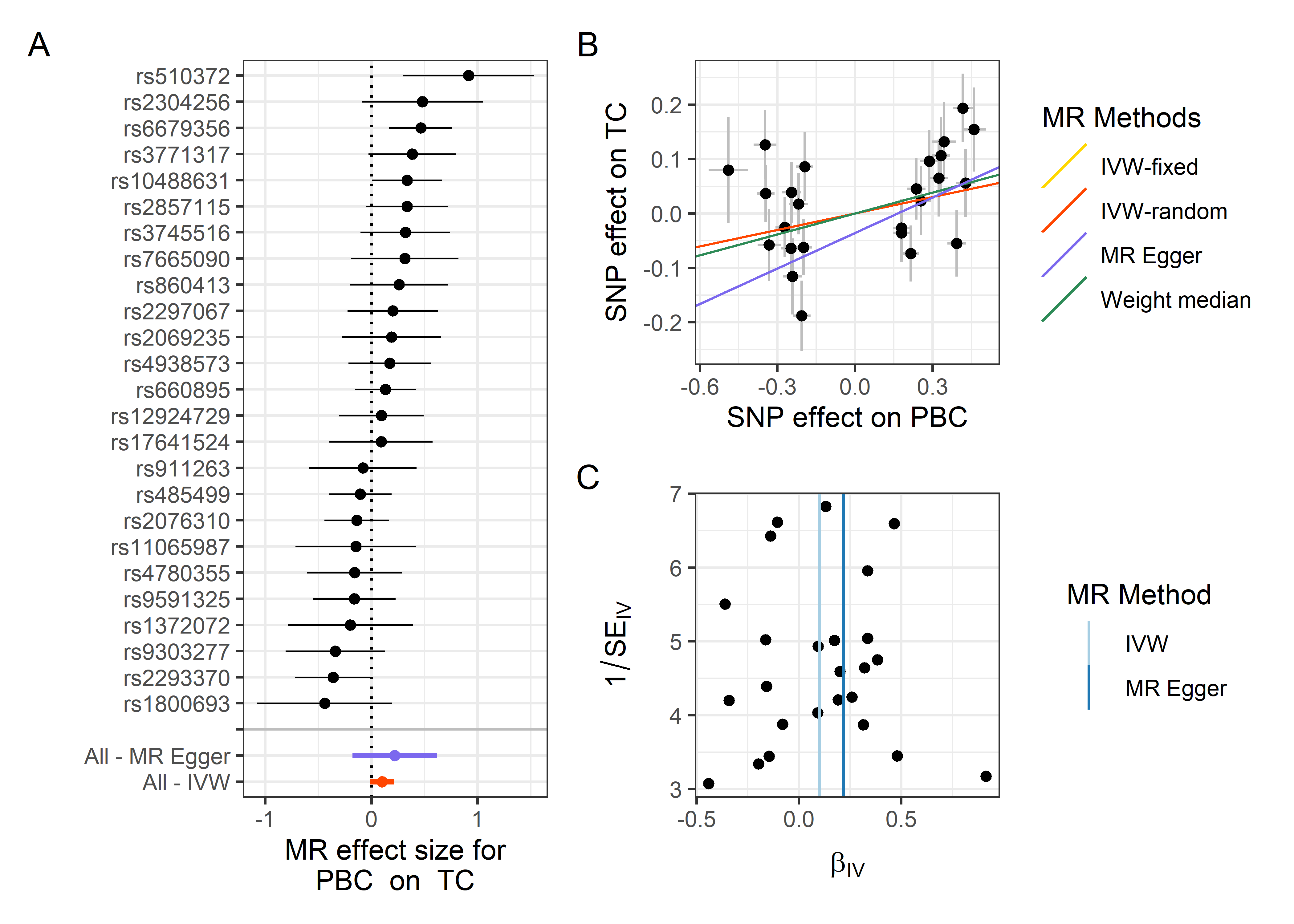

Supplement: Supplementary file 1 [file DataSheet1.ZIP › Supplementary figures/Fig.S4.tif]

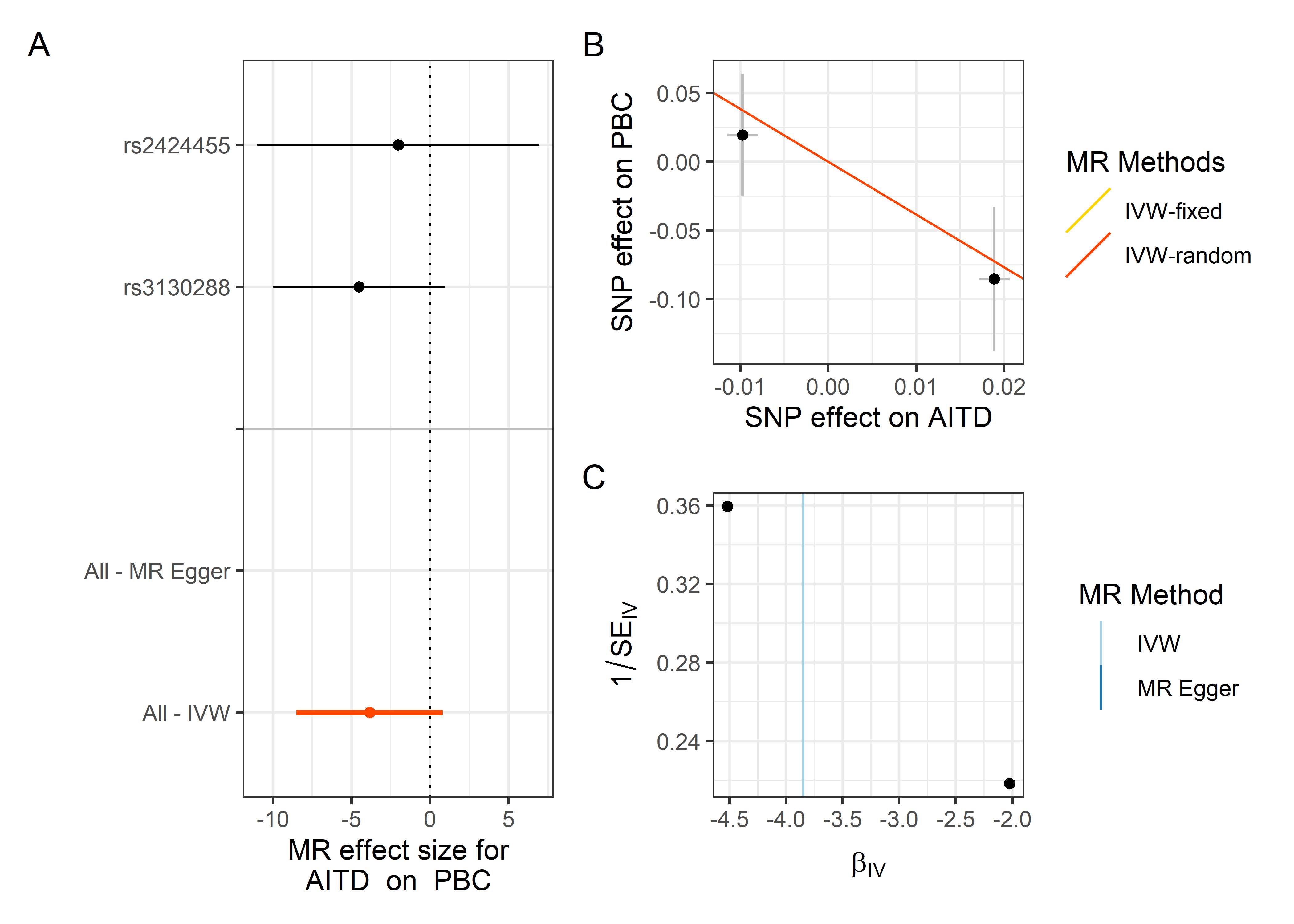

Supplement: Supplementary file 1 [file DataSheet1.ZIP › Supplementary figures/Fig.S5.tif]

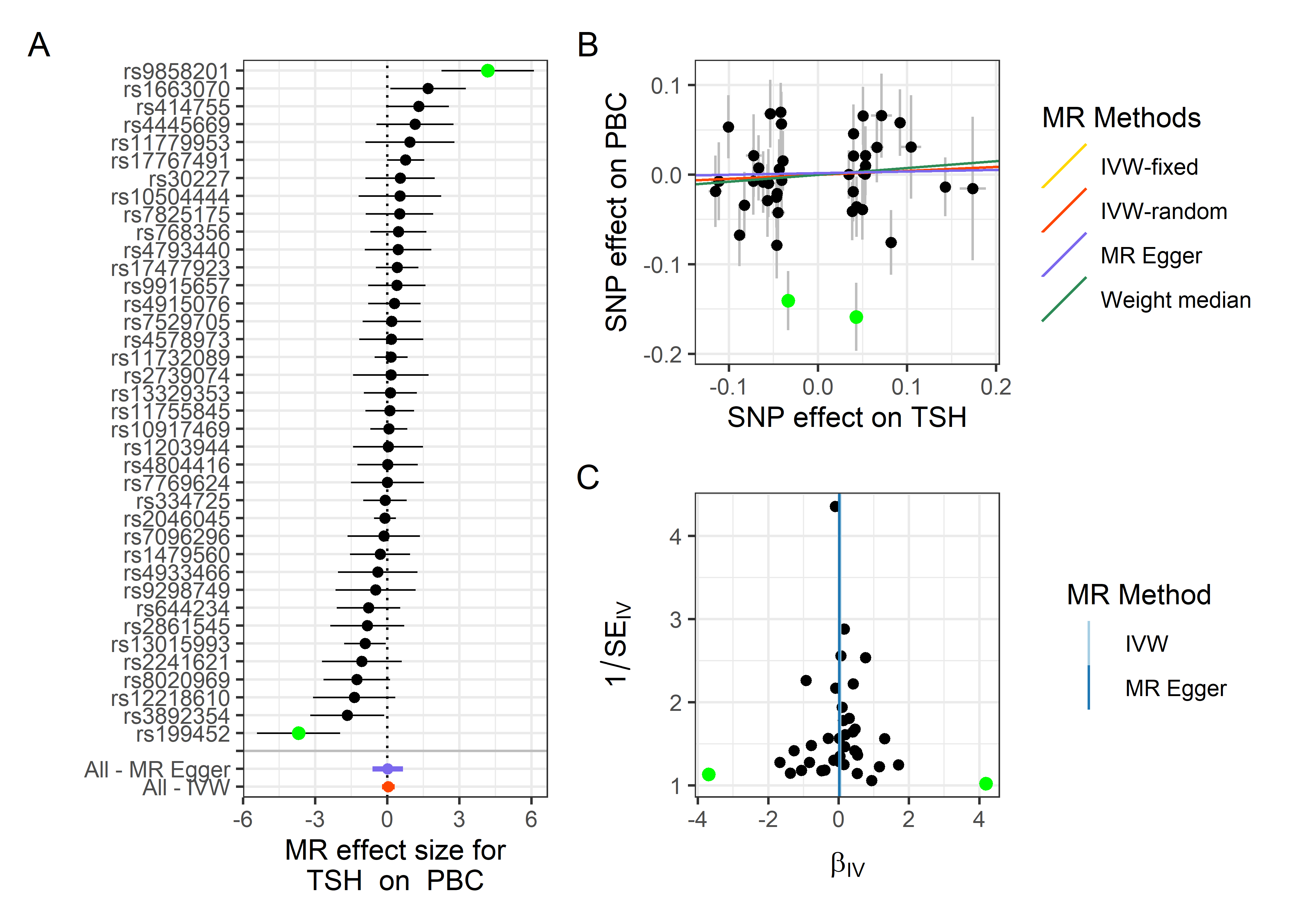

Supplement: Supplementary file 1 [file DataSheet1.ZIP › Supplementary figures/Fig.S6.tif]

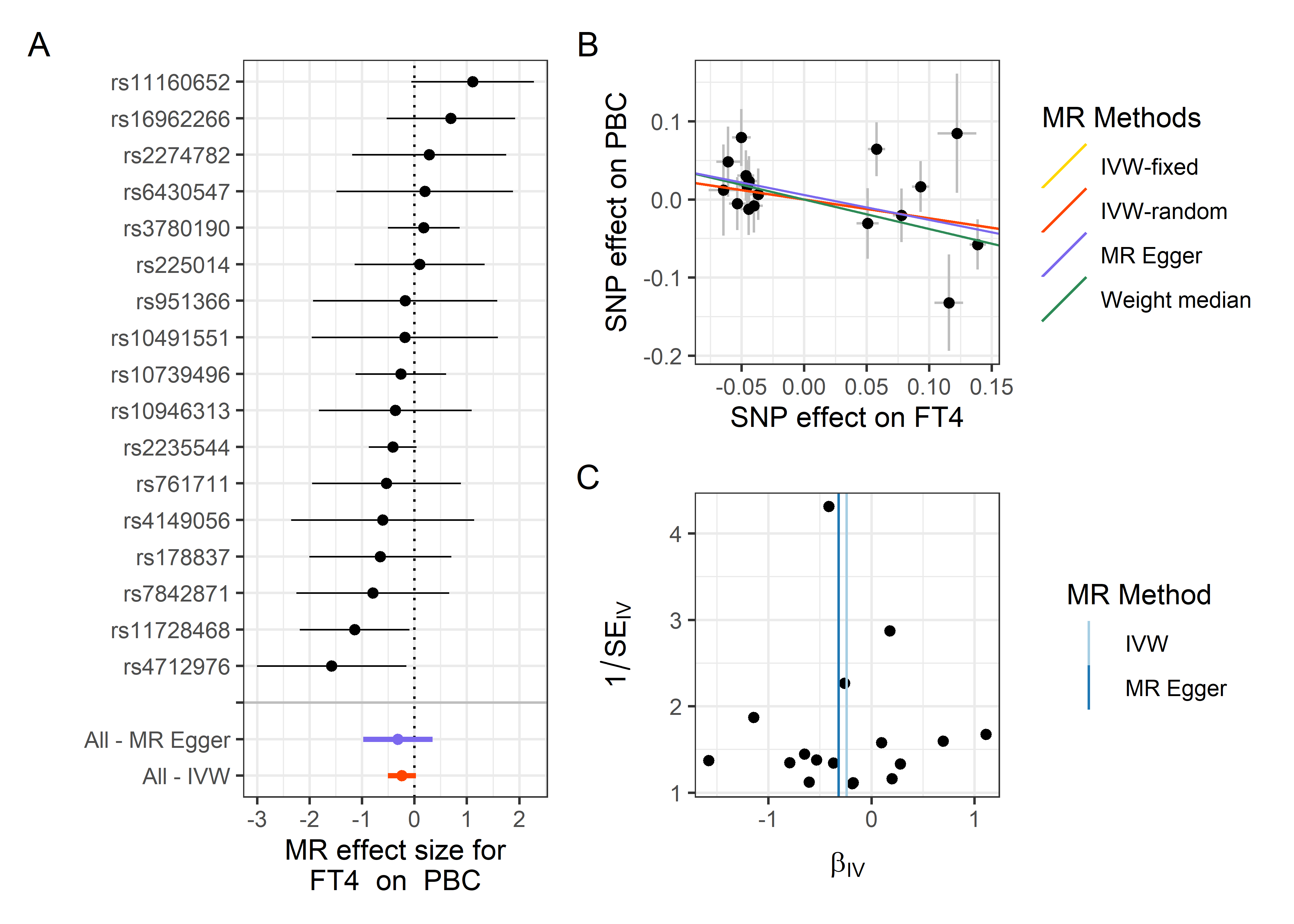

Supplement: Supplementary file 1 [file DataSheet1.ZIP › Supplementary figures/Fig.S7.tif]

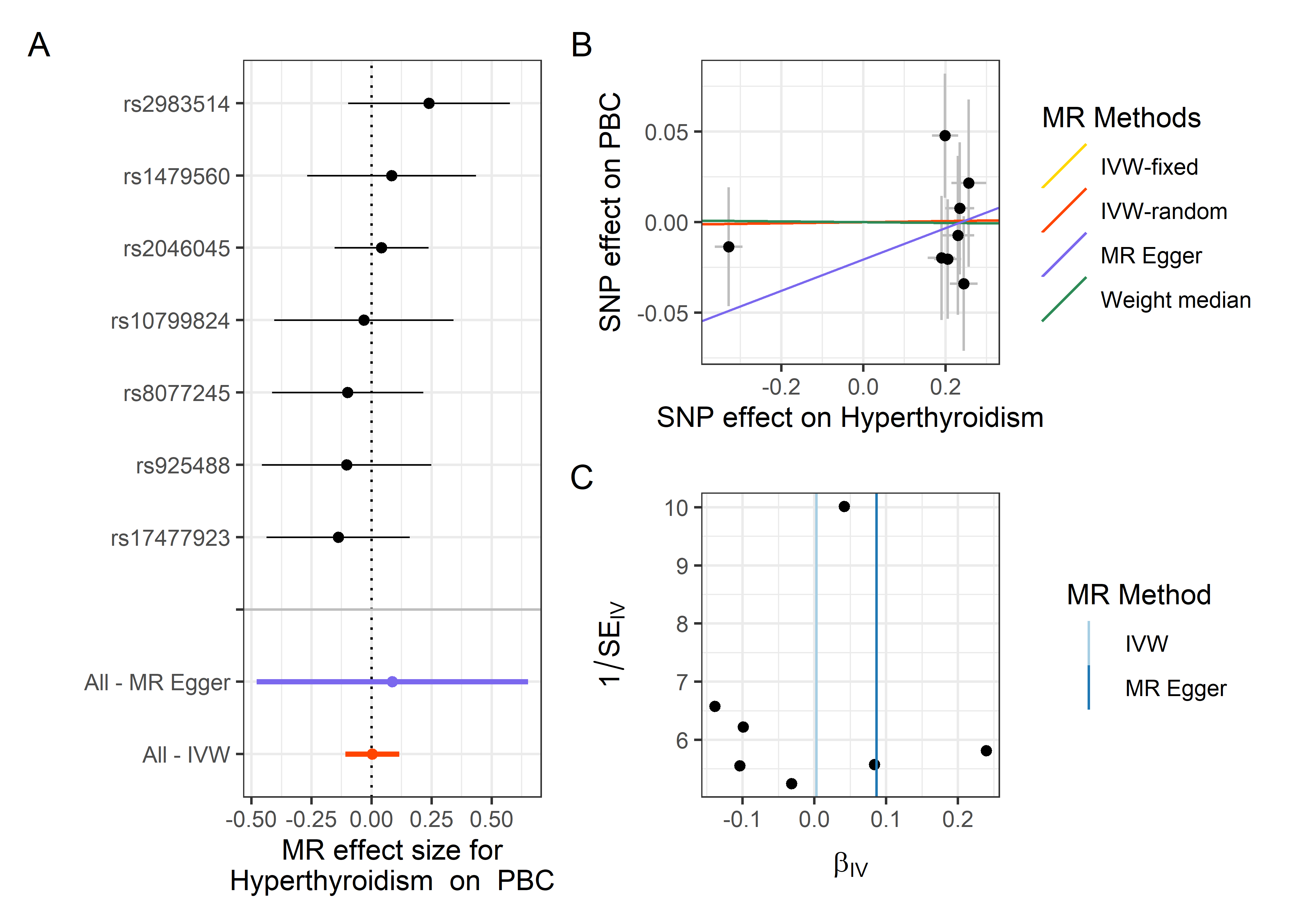

Supplement: Supplementary file 1 [file DataSheet1.ZIP › Supplementary figures/Fig.S8.tif]

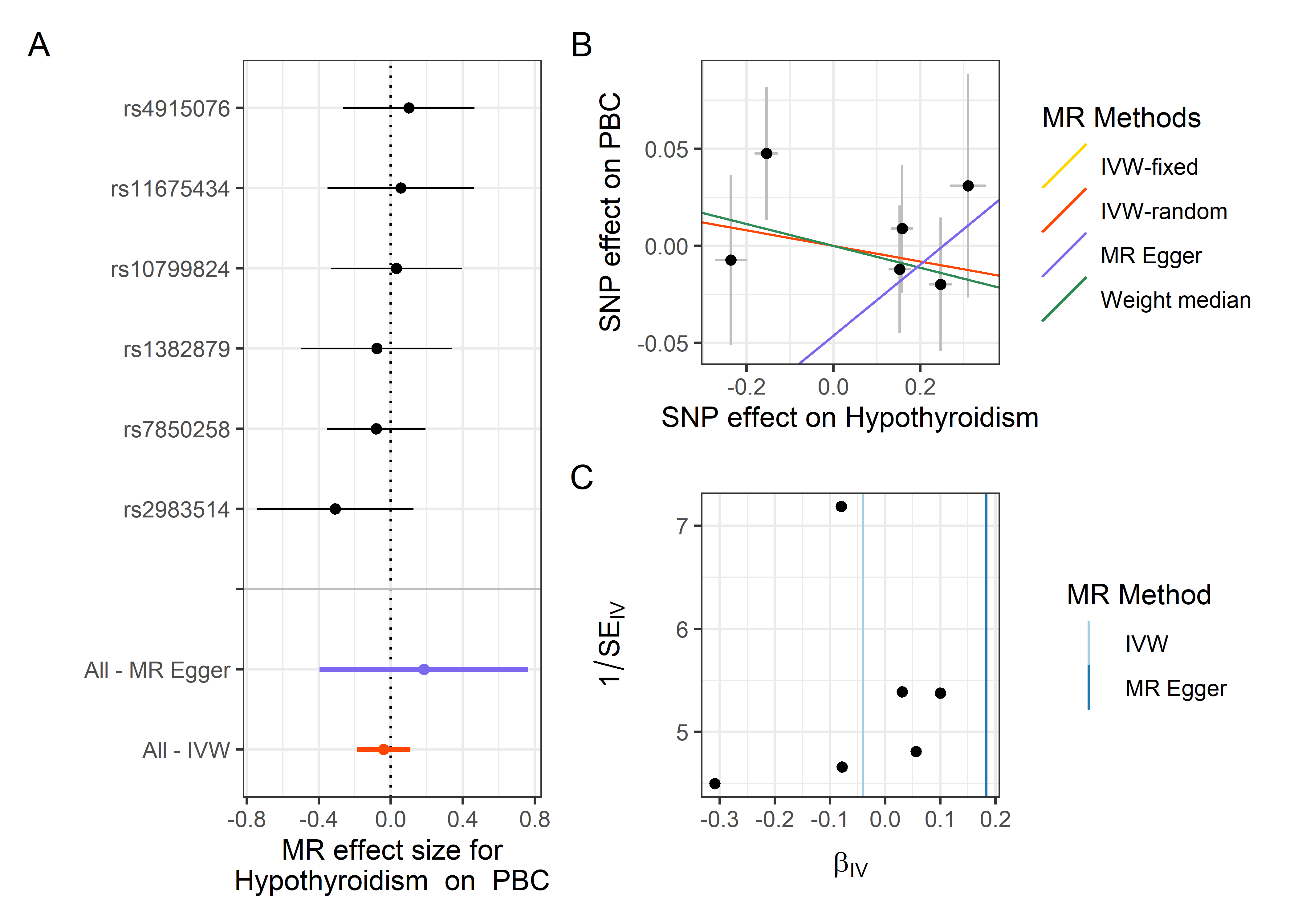

Supplement: Supplementary file 1 [file DataSheet1.ZIP › Supplementary figures/Fig.S9.tif]
